# DEUS: an R package for accurate small RNA profiling based on differential expression of unique sequences

**DOI:** 10.1093/bioinformatics/btz495

**Published:** 2019-06-22

**Authors:** Tim Jeske, Peter Huypens, Laura Stirm, Selina Höckele, Christine M Wurmser, Anja Böhm, Cora Weigert, Harald Staiger, Christoph Klein, Johannes Beckers, Maximilian Hastreiter

**Affiliations:** 1 Institute of Bioinformatics and Systems Biology, Helmholtz Zentrum München, Neuherberg 85764, Germany; 2 Department of Pediatrics, Dr. von Hauner Children’s Hospital, University Hospital, LMU Munich, München 80337, Germany; 3 Institute of Experimental Genetics, Helmholtz Zentrum München GmbH, Neuherberg 85764, Germany; 4 German Center for Diabetes Research (DZD), Neuherberg 85764, Germany; 5 Institute for Diabetes Research and Metabolic Diseases of the Helmholtz Zentrum München at the University of Tübingen, Tübingen 72076, Germany; 6 Chair of Animal Breeding, Technische Universität München, Wissenschaftszentrum Weihenstephan, Freising 85354, Germany; 7 Chair of Experimental Genetics, Technische Universität München, Wissenschaftszentrum Weihenstephan, Freising 85354, Germany; 8 Institute of Computational Biology, Helmholtz Zentrum München GmbH, Neuherberg 85764, Germany; 9 Chair of Genome-oriented Bioinformatics, Technische Universität München, Wissenschaftszentrum Weihenstephan, Freising 85354, Germany

## Abstract

**Summary:**

Despite their fundamental role in various biological processes, the analysis of small RNA sequencing data remains a challenging task. Major obstacles arise when short RNA sequences map to multiple locations in the genome, align to regions that are not annotated or underwent post-transcriptional changes which hamper accurate mapping. In order to tackle these issues, we present a novel profiling strategy that circumvents the need for read mapping to a reference genome by utilizing the actual read sequences to determine expression intensities. After differential expression analysis of individual sequence counts, significant sequences are annotated against user defined feature databases and clustered by sequence similarity. This strategy enables a more comprehensive and concise representation of small RNA populations without any data loss or data distortion.

**Availability and implementation:**

Code and documentation of our R package at http://ibis.helmholtz-muenchen.de/deus/.

**Supplementary information:**

Supplementary data are available at *Bioinformatics* online.

## 1 Introduction

A general approach to analyze small non-coding RNAs (sncRNA) data encompasses the evaluation of differential expression between conditions of interest. For this purpose, several software packages, such as miRDeep (Friedländer *et al.*, 2008), tDRmapper (Selitsky and Sethupathy, 2015), sRNAnalyzer (Wu *et al.*, 2017) and sRNAtoolbox (Rueda *et al.*, 2015), have been developed. A common step shared by these sncRNA profiling tools is the alignment of reads to a reference genome, followed by their annotation, feature count quantification and the subsequent statistical evaluation between experimental conditions (Anders *et al.*, 2013). However, the analysis of the expressed sncRNA populations poses several hurdles because short reads are more likely to map to multiple locations in the genome, or map to genomic coordinates that are not annotated and may deviate from the originating feature sequence due to editing and post-transcriptional processing steps. Here, we present our method that analyzes differential expression of unique sequences (DEUS) for profiling sncRNA sequence data without relying on read mapping.

## 2 Implementation

Our pipeline starts with the identification of unique reads in each of the input FASTQ files to generate a typical RNA-seq count matrix, but utilizing the actual read sequence instead of the gene feature as identifier. The count table is then used as input for DESeq2 (Love *et al.*, 2014) analysis to calculate statistically significant read count differences between samples from different experimental conditions. Adjusted *p*-values for the differentially expressed (DE) unique sequences are calculated according to the Independent Hypothesis Weighting method (Ignatiadis *et al.*, 2016) using the means of normalized counts as covariate. DE unique sequences are subsequently annotated by BLASTn (Altschul *et al.*, 1990) searches against user defined BLAST databases. Subsequently, the CD-Hit clustering algorithm (Fu *et al.*, 2012; Li and Godzik, 2006) is applied to classify significant DE reads into subgroups of similar sequences based on the percentage of sequence identity and the length of the overlapping sub-sequences as defined by the user. This additional information can be used to inspect significant sequences in groups that indicate similar biological origin. Finally, a comprehensive summary table is generated by combining results from differential expression analysis, BLASTn annotation and cluster assignment (Supplementary Table S1). To easily explore the content of the table the user can define an individual set of terms that represent feature classes of interest. The given terms will be integrated as columns each containing the number of BLAST hits that match the corresponding term. DEUS also automatically generates plots to visualize the expression intensities versus fold changes of the identified sequences and the distance of the expression profiles of the samples in analysis. Additionally, we implemented an extended approach that performs clustering and summarizes sequence counts prior to differential expression analysis to provide further insights on a more general level (see Supplementary Material). We implemented each of the described steps as customizable functions in the R package DEUS. This modular design allows the user to customize our pipeline, tailored to the specific needs of the project.

## 3 Discussion

**Fig. 1.**
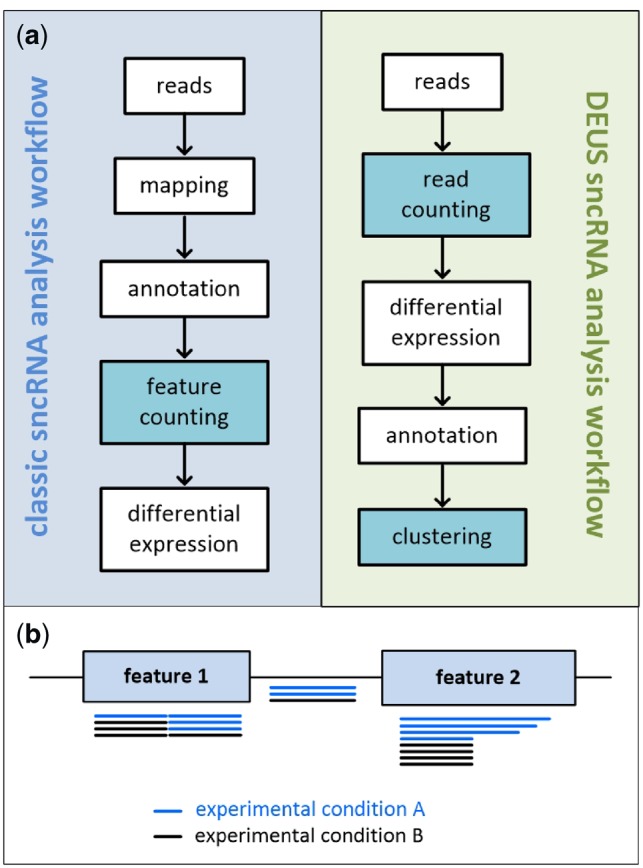
Major differences between mapping-based and DEUS small RNA profiling strategies. (**a**) Schematic representation of the workflow of mapping-based pipelines compared with DEUS. (**b**) Schematic representation of scenarios that result in data distortion or data loss when applying mapping-based sncRNA profiling strategies. Mapping-based workflows ignore reads that map to non-annotated genome regions (depicted as reads between the two features) and foster data distortion as variant-specific read counts are usually summed up during subsequent feature counting even if these reads align at different spatial coordinates of the same genomic feature (depicted as reads mapped to feature 1) or exhibit discrete variations in nucleotide sequence or sequence length (depicted as reads mapped to feature 2)

In accordance with Johnson *et al.* (2016), we found that sncRNA datasets from various mouse and human biomaterials are plagued by substantial amounts of multi-mapping reads (61.5 ± 20.1%) and noticeable amounts (44.7 ± 17.2%) of reads that map to regions of the genome that are not annotated (Supplementary Fig. S1 and Supplementary Table S2). Consequently, it requires dedicated methods that account for these issues. DEUS deviates from mapping-based small RNA profiling methods in several aspects (Fig. 1a). As DEUS is not relying on mapping it facilitates sncRNA profiling even when a reference genome is not available. Further, it includes all reads in analysis even those that were mapped to loci lacking feature annotation and those that cannot be mapped, for example, due to extensive RNA editing events (Fig. 1b). DEUS circumvents the challenge of correctly assigning multi-mapping reads to their originating feature by representing multiple putative mapping positions by multiple annotations per unique sequence. Due to the use of unique sequences, DEUS inherently detects discrete sequence or length variations. The information about sequence variations would otherwise be hidden in read counts grouped on feature-level or lost if varying reads could not be mapped. To allow feature-based result interpretation despite sequence-based data analysis, DEUS clusters highly similar sequences (Fig. 1a). This compression of resulting DE sequences to sequence clusters reduces the number of result entities in a range from about 40% up to 80%. In combination with the extended differential expression analysis, the use of sequence clusters improves the overall signal detection power and provides a second data perspective that includes single sequence and cluster-level analysis.

In summary, DEUS provides an unprecedented way to profile and visualize sncRNA data. DEUS clearly diverges from mapping-based analysis strategies, hampered by substantial data loss and distortion of feature counts. We believe that our DEUS pipeline considerably improves the analysis of sncRNA-seq data, being applicable in various existing pipelines and returning intuitively interpretable results.

## Funding

The work was supported by funding of The Leona M. and Harra B Helmsley Charitable Trust, the Care-for-Rare Foundation, BMBF (PID-NET, 01GM1517A) and by grants of the DZD - German Center for Diabetes Research and the Helmholtz Alliance AMPro to JB.

*Conflict of Interest*: none declared.

## Supplementary Material

btz495_Supplementary_Data
