# Supplementary material for: DEUS: an R package for accurate small RNA profiling based on differential expression of unique sequences
Source: Bioinformatics. 2019 Jun 22;35(22):4834–6. doi: 10.1093/bioinformatics/btz495 (PMC6853685; doi:10.1093/bioinformatics/btz495)
Supplement: btz495_Supplementary_Data [file btz495_supplementary_data.zip › btz495-suppl_data/DEUS_Supplement.pdf]

DEUS Supplementary Material

Multi-mapping analysis of biomaterials

DEUS differs from most sncRNA analysis tools as it does not rely on read mapping and conducts annotation after differential expression analysis on sequence-based read counts. Using 150 samples from various mouse and human biomaterials, we also provide evidence that data distortion and data loss are considerable when using a mapping-based strategy. Interestingly, we found that the percentage of multi-mapping reads is significantly higher in biomaterials such as plasma, sperm and exosomes when compared to somatic cell types and colorectal cancer cell lines (78.1 ± 7.4% vs 44.9 ± 12.4%, respectively; One-way ANOVA, post-hoc Bonferroni, $p<0.001$) indicating that mapping-based inflicted read count distortion may be more considerable when dealing with such biological materials (Supplementary Figure 1).

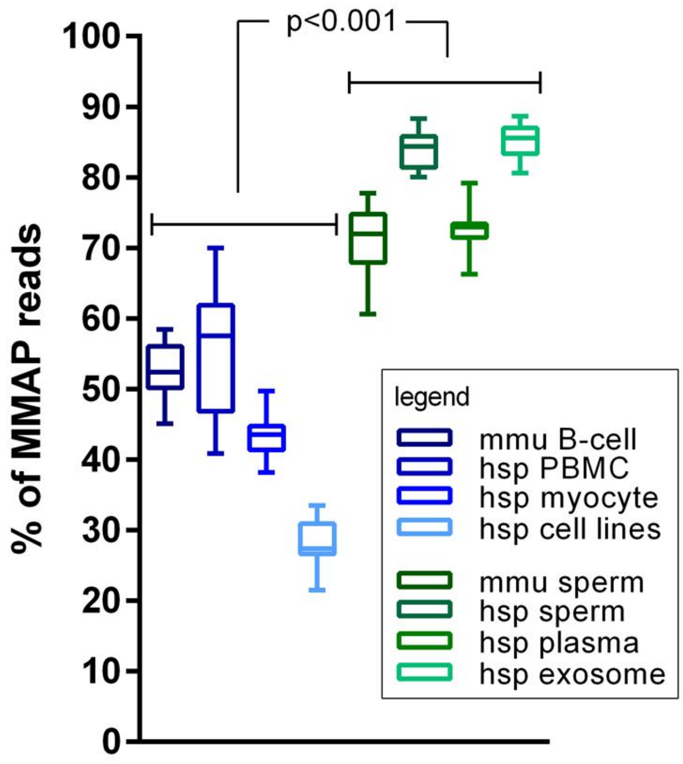

Supplementary Figure 1: Multi-mapping fractions across different cell types and lines.
The abundance of multi-mapped reads in sperm, plasma and exosomes is significantly higher as compared to somatic cell types and cell lines. Box and whiskers plot showing the percentage of multi-mapped (MMAP) reads in mouse (mmu) B-cells, human (hsp) PBMCs, human myocytes, human colorectal cancer cell lines, mouse and human sperm, human plasma and exosomes released from human colorectal cancer cell lines.

Extended differential expression analysis using sequence clusters

In order to assess the potential loss of power that could occur when unique sequences are analyzed individually (compared to the more usual approach where reads are summarized for each genomic feature), we implemented an adjusted DEUS workflow, dedicated for combining both approaches.

In addition to the main idea and method presented in our manuscript, unique sequences are grouped into clusters with high sequence similarity. Subsequently, sequence counts are summed for each sequence cluster. The resulting count matrix is used for DE analysis producing an additional list of differentially expressed sequence clusters. Finally, the result is integrated into the single-sequence analysis to allow a direct comparison of both methods.

We applied this modified workflow to publicly available sncRNA sequencing data derived from human colorectal cancer cell lines and their released exosomes (Cha, et al., 2015) to analyze differential expression between cells and exomes of the DKs-8 cell line.

Overall, we detected 184,612 significant sequences and 115,875 significant sequence clusters. The significant clusters include a total of 3,444,231 unique sequences, adding a large amount of 3,269,805 sequences which were not detected as being differentially expressed on a single sequence level. As a result, also 29.37% (34,034) of the significant clusters include no significant sequences. Subsequently, we investigated the reason for the large number of non-significant sequences in significant clusters. For 96.53% (3,156,250) of the cases, the observation can be explained by very low expression values that lead to the exclusion of those sequences prior to the differential expression analysis in the single sequence approach. The remaining 3.47% (113,555) had no significant differential expression on a single sequence level. Conversely, we found 7,132 sequences that are significant on a single sequence level and are part of in non-significant clusters.

As expected, these findings show that the approach of clustering sequences before testing for differential expression can identify several additional targets that might be interesting for further investigations. Nevertheless, it has to be noted that although the overall power for detecting differential expression is increased, it is based on summarizing counts on a common feature, similar to mapping-based methods. Therefore, this comes with all common drawbacks which were discussed earlier.

By applying the presented workflow which performs single and cluster-level analysis, an appropriate way was found to account for both ideas.

Material and methods

Library preparation Total RNA served as input material for small RNA library preparation using NEBNext Small RNA Library Prep Set for Illumina (New England BioLabs) according to the manufacturer's guidelines. Following a brief denaturation step, 3′ adapters were ligated to the input RNA for 1 hour, followed by hybridization of the reverse transcription primers and subsequent ligation of the 5′ adapters. Next, reverse transcription was performed using ProtoScript II for 1 hr at 50°C and the sequence and index primers were added by PCR amplification for 11-15 cycles using LongAmp Taq 2× master according to the manufacturer's recommendations.

Illumina 2500 HiSeq sequencing The libraries were sequenced on a HiSeq2500 (Illumina inc., San Diego, CA, USA) using HiSeq Rapid v2 chemistry (Illumina). Raw data was collected by the Illumina HiSeq Control Software (version 2.2.58). Illumina Real-Time Analysis tool (version 1.18.64) was used for image analysis and base calling. The single-end sequence reads with 50 base pair read length were demultiplexed and FASTQ files were generated with CASAVA BCL2FASTQ Conversion Software (version 1.8.3).

Read trimming Trim Galore (v0.0.4, http://www.bioinformatics.babraham.ac.uk/projects/trim_galore/) was used to trim reads of all analyzed data sets in two steps. In the first step, all adapters were removed and only reads shorter than the maximum sequencing length were kept. In the second step, reads were trimmed for a minimum quality threshold of 20.

Read mapping To compute the proportion of reads mapping to multiple genomic locations, we used STAR v2.5.2a (Dobin, et al., 2012) to map the trimmed reads to the primary assembly of the human reference genome GRCh38 for human data sets and to the mouse reference genome GRCm38 for mouse data sets. STAR was configured to match at least 16 nucleotides with a maximum of 5% mismatches over the mapped length having the splicing function switched off.

Read annotation We applied our DEUS pipeline to all data sets and computed the number of reads that had no BLAST hit. We manually compiled available sncRNA databases to generate comprehensive BLAST databases. For human data sets we included the Ensembl CDS and ncRNA databases (Zerbino, et al., 2018), DASHR, the database of small human noncoding RNAs (Leung, et al., 2016), and the RetrogeneDB (Kabza, et al., 2014). Overall, the human database comprises a total of 193,634 sequences. For mouse data sets, Ensembl CDS and ncRNA databases (Zerbino, et al., 2018), miRBase (Kozomara and Griffiths-Jones, 2014), RetrogeneDB (Kabza, et al., 2014), piRNABank (Sai Lakshmi and Agrawal, 2008) and GtRNAdb (Chan and Lowe, 2009; Chan and Lowe, 2016) were used, resulting in a total of 126,153 sequences. We used the NCBI BLASTn 2.6.0+ (Altschul, et al., 1990) requiring 100% sequence identity to annotate sequences that we identified in our data sets.

Read clustering To compute the compression of differentially expressed sequences to sequence clusters and to cluster the complete sequence count table in the context of the sequence cluster approach, we used the

CD-Hit clustering tool (CD-HIT version 4.7) (Fu, et al., 2012; Li and Godzik, 2006). Clustering was performed in accurate mode with a sequence identity threshold of 0.9, a length difference cutoff of 0.9 and a word length of 9.

Statistical analysis The statistical analysis shown in Supplementary Figure 1 was performed using GraphPad Prism (GraphPad Software, La Jolla California USA). Data represent mean ± SD. The box and whiskers graphs show the 25th to 75th percentiles, with whiskers extending from the smallest up to the largest value. The line plotted in the middle of the box represents the median. One-way ANOVA, post-hoc Bonferroni multiple comparisons test, with a confidence interval of 99.9% was used to evaluate statistical significance between groups, showing the least significant p-value in the comparison between samples belonging to somatic cell types and colorectal cell lines on one hand and carrier sncRNA samples such as plasma, sperm and exosomes on the other.

Data availability We collected a total of 150 small RNA data samples. sncRNA sequencing data derived from human colorectal cancer cell lines (n=9) and their released exosomes (n=9) (Cha, et al., 2015) was obtained from NCBI GEO (Barrett, et al., 2011; Barrett, et al., 2013), dataset accession number: GSE67004, ID:200067004. Small RNA sequencing data from human plasma (n=19), human blood mononuclear cells (PBMCs, n=16), human myocytes (n=20), human sperm (n=14), mouse sperm (n=17) and mouse B-cells (n=46) are part of active research projects and are available on reasonable request. Please direct your data requests to HS (harald.staiger@med.uni-tuebingen.de) for human plasma and human blood mononuclear cells, to CW (cora.weigert@med.uni-tuebingen.de) for human myocytes, to JB (beckers@helmholtz-muenchen.de) for human and mouse sperm and to Marcin Lyszkiewicz for mouse B-cells (marcin.lyszkiewicz@med.uni-muenchen.de).

Supplementary Table 1: DEUS output files

File name: Supplementary Table 1.xlsx

Example of a typical DEUS output file with a focus on two sequence clusters. The output file lists the actual sequence, sequence ID, log2 fold change, p-value, IHW p-value, average read counts for each experimental group, standard deviation (SD), cluster ID, sequence length, blast e-value and the number of annotation hits for several user specified small RNA species classes in consecutive columns as well as a comma separated feature list. The ten sequences belonging to cluster 807 display several sequence variations of miR-340, some of which are regulated in the opposite direction when compared to the original miR sequence. The four sequences belonging to cluster ID 300 are frequent multi-mapping reads that are annotated both as piRNAs and tRNAs with 100% sequence identity.

Supplementary Table 2: Overview of multi-mapped and non-annotated reads

File name: Supplementary Table 2.xlsx

The table lists the number of reads, their average length after adapter trimming, the total number of input reads, the total number of unique reads, the fraction of unique reads, the fraction of uniquely mapped read as well as the percentage of multi-mapped and non-annotated reads for each of the analyzed RNA-seq samples. The first tab shows the overview of all samples, whereas the other tabs show these values for each of the individual data sets.

Acknowledgement

We thank CMW for conducting high-throughput sequencing and LS, SH, AB, CW, HS, CK and JB for providing data sets for the validation of the pipeline. We further thank Natalia Zietara and Marcin Lyszkiewicz in the group of Christoph Klein at the Department of Pediatrics at the Dr. von Hauner Children's Hospital for providing RNA-seq data of B-cells.

References

Altschul, S.F., Gish, W., Miller, W., Myers, E.W. and Lipman, D.J. Basic local alignment search tool. J. Mol. Biol. 1990;215:403-410.

Barrett, T., *et al.* NCBI GEO: archive for functional genomics data sets--10 years on. *Nucleic Acids Res* 2011;39(Database issue):D1005-1010.

Barrett, T., *et al.* NCBI GEO: archive for functional genomics data sets--update. *Nucleic Acids Res* 2013;41(Database issue):D991-995.

Cha, D.J., *et al.* KRAS-dependent sorting of miRNA to exosomes. *eLife* 2015;4:e07197.

Chan, P.P. and Lowe, T.M. GtRNAdb: a database of transfer RNA genes detected in genomic sequence. *Nucleic Acids Res* 2009;37(Database issue):D93-97.

Chan, P.P. and Lowe, T.M. GtRNAdb 2.0: an expanded database of transfer RNA genes identified in complete and draft genomes. *Nucleic Acids Res* 2016;44(D1):D184-189.

Dobin, A. et al. "STAR: ultrafast universal RNA-seq aligner." *Bioinformatics* vol. 2012;29,1: 15-21.

Fu,L., Niu,B., Zhu,Z., Wu,S. and Li,W. CD-HIT: accelerated for clustering the next-generation sequencing data.Bioinformatics 2012;28 (23), 3150–3152

Kabza, M., Ciomborowska, J. and Makalowska, I. RetrogeneDB--a database of animal retrogenes. *Molecular biology and evolution* 2014;31(7):1646-1648.

Kozomara, A. and Griffiths-Jones, S. miRBase: annotating high confidence microRNAs using deep sequencing data. *Nucleic Acids Res* 2014;42(Database issue):D68-73.

Li,W. and Godzik,A. Cd-hit: a fast program for clustering and comparing large sets of protein or nucleotide sequences. Bioinformatics (Oxford, England) 2006;22 (13), 1658–1659

Leung, Y.Y., *et al.* DASHR: database of small human noncoding RNAs. *Nucleic Acids Res* 2016;44(D1):D216-222.

Sai Lakshmi, S. and Agrawal, S. piRNABank: a web resource on classified and clustered Piwi-interacting RNAs. *Nucleic Acids Res* 2008;36(Database issue):D173-177.

Zerbino, D.R., *et al.* Ensembl 2018. *Nucleic Acids Res* 2018;46(D1):D754-d761.
